# Tuberculosis and HIV—An Update on the “Cursed Duet” in Children

**DOI:** 10.3389/fped.2019.00159

**Published:** 2019-04-25

**Authors:** Samantha H.-L. Fry, Shaun L. Barnabas, Mark F. Cotton

**Affiliations:** Family Centre for Research with Ubuntu (FAM-CRU), Department of Paediatrics and Child Health, Faculty of Medicine and Health Sciences, Stellenbosch University, Stellenbosch, South Africa

**Keywords:** tuberculosis, HIV, childhood TB, TB/HIV co-infection, TB/HIV co-therapy

## Abstract

HIV and tuberculosis (TB) often occur together with each exacerbating the other. Improvements in vertical transmission prevention has reduced the number of HIV-infected children being born and early antiretroviral therapy (ART) protects against tuberculosis. However, with delayed HIV diagnosis, HIV-infected infants often present with tuberculosis co-infection. The number of HIV exposed uninfected children has increased and these infants have high exposure to TB and may be more immunologically vulnerable due to HIV exposure *in utero*. Bacillus Calmette-Guérin (BCG) immunization shortly after birth is essential for preventing severe TB in infancy. With early infant HIV diagnosis and ART, disseminated BCG is no longer an issue. TB prevention therapy should be implemented for contacts of a source case and for all HIV-infected individuals over a year of age. Although infection can be identified through skin tests or interferon gamma release assays, the non-availability of these tests should not preclude prevention therapy, once active TB has been excluded. Therapeutic options have moved from isoniazid only for 6–9 months to shorter regimens. Prevention therapy after exposure to a source case with resistant TB should also be implemented, but should not prevent pivotal prevention trials already under way. A microbiological diagnosis for TB remains the gold standard because of increasing drug resistance. Antiretroviral therapy for rifampicin co-treatment requires adaptation for those on lopinavir-ritonavir, which requires super-boosting with additional ritonavir. For those with drug resistant TB, the main problems are identification and overlapping toxicity between antiretroviral and anti-TB therapy. In spite of renewed focus and improved interventions, infants are still vulnerable to TB.

## Summary

Tuberculosis (TB) is a leading cause of morbidity and mortality in both adults and children across the globe. The causative agent, *Mycobacterium tuberculosis* (*M.tb*), causes more deaths than any other infectious agent. Human Immunodeficiency Virus (HIV) coinfection contributes greatly to the global burden of TB, particularly in sub Saharan Africa, where the prevalence of both diseases is high. The ambitious goal to eradicate TB, as set by the World Health Organization (WHO), faces multiple challenges, particularly for childhood TB. In the HIV/TB co-infected child, the risk of infection and disease is increased, diagnosis is challenging, and treatment involves high medication burden and complex drug interactions. However, national TB programs highlighting early identification of childhood case contacts, adequate and appropriate preventative therapy, novel strategies, and advancements in TB diagnostics and drug therapy, and early initiation of antiretroviral therapy are all positive steps toward achieving this goal.

## Introduction

Since the identification of *Mycobacterium tuberculosis* (*M.tb*) as the cause of tuberculosis (TB) in March 1882 (1), *M.tb* remains one of most lethal human pathogens, causing more deaths than any other infectious agent (2). In 2017, there were an estimated 10 million incident TB cases globally, ~133 cases/100,000. Children below 15 years of age contributed 10% of cases, mostly in South East Asia (35.8%), Africa (29%), and the Western Pacific Region (20.5%). In Africa this coincided with the HIV pandemic (3). Key data shown in Box 1.

Box 1 **Key data: 2017.**

**Table:** 

	**TB (3)**	**HIV (10)**
Global incident cases	10 million	1.8 million ~5,000 new infections per day
Incident cases in children <15 years	1 million	160,000
Number of new TB cases among HIV+ people		900,000
Number of people living with HIV	–	36.9 million
Number of children living with HIV	–	2.1 million
Treatment coverage	64%	59%
Number of people who developed drug resistant TB	558,000	
Number of people estimated to have TB infection	1.7 billion (23% of the world's population	–
Number of TB deaths among HIV negative people	1.3 million	–
Number of TB deaths among HIV + people		370,000
Number of people who died from HIV-related illnesses		940,000

In 1993 the World Health Organization (WHO) declared TB a global emergency and in 2016 implemented a new END TB strategy to eradicate TB by 2035, aligning with the sustainable development goals (SDG) (3).

The acquired immunodeficiency syndrome (AIDS) was first recognized in 1981 in adults and in infants in 1982 (4, 5). HIV was identified as the cause of AIDS in 1983 (6). Shortly thereafter, systematic studies establishing the enormous scale of AIDS and the origin of HIV began in Africa (7).

## TB Disease and HIV

Chretién, recognizing that TB was more severe in immunosuppressed HIV-infected (HIV+) adults than the general population, introduced the term “the cursed duet” in 1990 (8). HIV infection increases the progression of TB infection to disease 30-fold and worsens disease severity (9). Globally, 36.7 million people are HIV+, including 2.1 million children <15 year of age. Of the 1.8 million new HIV infections in 2016, 160,000 were children (10). An estimated 10% of the 1 million incident TB cases and 22% of the 374,000 deaths due to TB occurred in the HIV+ population (2) (Box 1). Thus, for TB eradication, TB/HIV co-infection must be addressed.

Similarities between HIV and TB are outlined in Table 1. Both conditions are immunosuppressive and cause a decline in CD4+ T-cell count (11). This decline of specific CD4+ T cells in both peripheral and pulmonary TB may facilitate progression from latent to active disease (12). Additionally, HIV impairs apoptosis of alveolar macrophages, a vital component of the innate response to *M.tb* (9).

**Table 1:** TB and HIV—commonalities.

**Factor**	**For HIV and TB**	**Comments**
Transmission	Both can be transmitted vertically	HIV: vertical transmission main route for HIV, begins *in utero* and extends through breast feeding TB: vertical transmission less common, peri- and post-natal transmission through close contact
	Prevention	Detection and treatment in mothers can prevent transmission
		Post-exposure prophylaxis effective
Epidemiology	Associated with poverty, overcrowding and poor access to health care Both occur within families and households	
Presenting symptoms and signs	Failure to thrive common in both	
	Respiratory symptoms common	
	Chronic lung disease including bronchiectasis and cavities common	
	Gastrointestinal disease occurs	
	Chest radiology often abnormal	
Disease course	More rapid progression in children. Youngest infants are most vulnerable	
Immunology	CD4+ depletion	
Correlates of severe disease	Organism load correlates with disease severity	
Treatment	Combination therapy with at least 3 drugs	
	Adherence monitoring essential for successful therapy	
	Immune reconstitution inflammatory syndrome (IRIS) can occur when commencing treatment for each	TB and BCG IRIS common in HIV + children commencing ART
Vaccines	Currently inadequate protection by specific vaccines	BCG prevents disseminated TB and TB meningitis in infants

Both culture-positive and -negative TB are common in HIV+ children (13–15). In a South African study from the late 1990's that predated availability of antiretroviral therapy (ART), nearly half (48.9%) of children hospitalized with pulmonary TB were HIV+. They had more cavitation and miliary disease than uninfected children (13). In a similar Nigerian study, 30% of children with TB were also HIV+ (14).

In 2012, Wiseman et al proposed a classification of childhood TB based on disease severity within an organ (mainly the lung), degree of dissemination beyond one organ or anatomical site and the extent of host control prior to introducing anti-TB therapy (16). Using this classification in culture-positive children below 12 months of age, severe disease occurred in 71% of the cohort, with similar rates in HIV+ and uninfected infants (16).

## The Impact of Improved Vertical HIV Transmission Prevention

Since implementing and subsequently improving programs to prevent mother to child transmission of HIV, fewer HIV+ infants are being born. The large-scale ART rollout, which began in 2002, and the implementation of the WHO's Option B (ART in pregnancy and during breast feeding) program in 2013 (17), upscaling to Option B+ (uninterrupted ART during pregnancy and thereafter) in 2016, has decreased the early South African perinatal HIV transmission rate to 0.9% (18). However, by 18 months of age the cumulative transmission rate was 4.4%, most likely due to breast-feeding. As these data preceded wide scale implementation of Option B/B+ (19), the cumulative transmission rate has likely decreased. Another factor may be insufficiently sensitive diagnostic tests related to very low HIV reservoirs from increased ART exposure (20, 21). Regardless of cause, delays in diagnosing HIV in infants will increase symptomatic HIV and TB.

## The HIV-Exposed Uninfected Child

Due to progress in prevention mother to child HIV transmission, there are now more HIV exposed uninfected (HEU) children (18). This population still has higher rates of morbidity and mortality, particularly from infectious diseases than HIV unexposed infants (22, 23). Impaired immunity and increased maternal or household TB and ART exposure are potential contributory factors to this elevated risk. HEU infants have altered cell-mediated immunity, including impaired T-cell maturation, and hypo- and hyper-responsive T-cell activation (22). These immune changes may be mediated by fetal HIV exposure as they are absent in mothers established on ART at conception (24).

**Figure 1:**
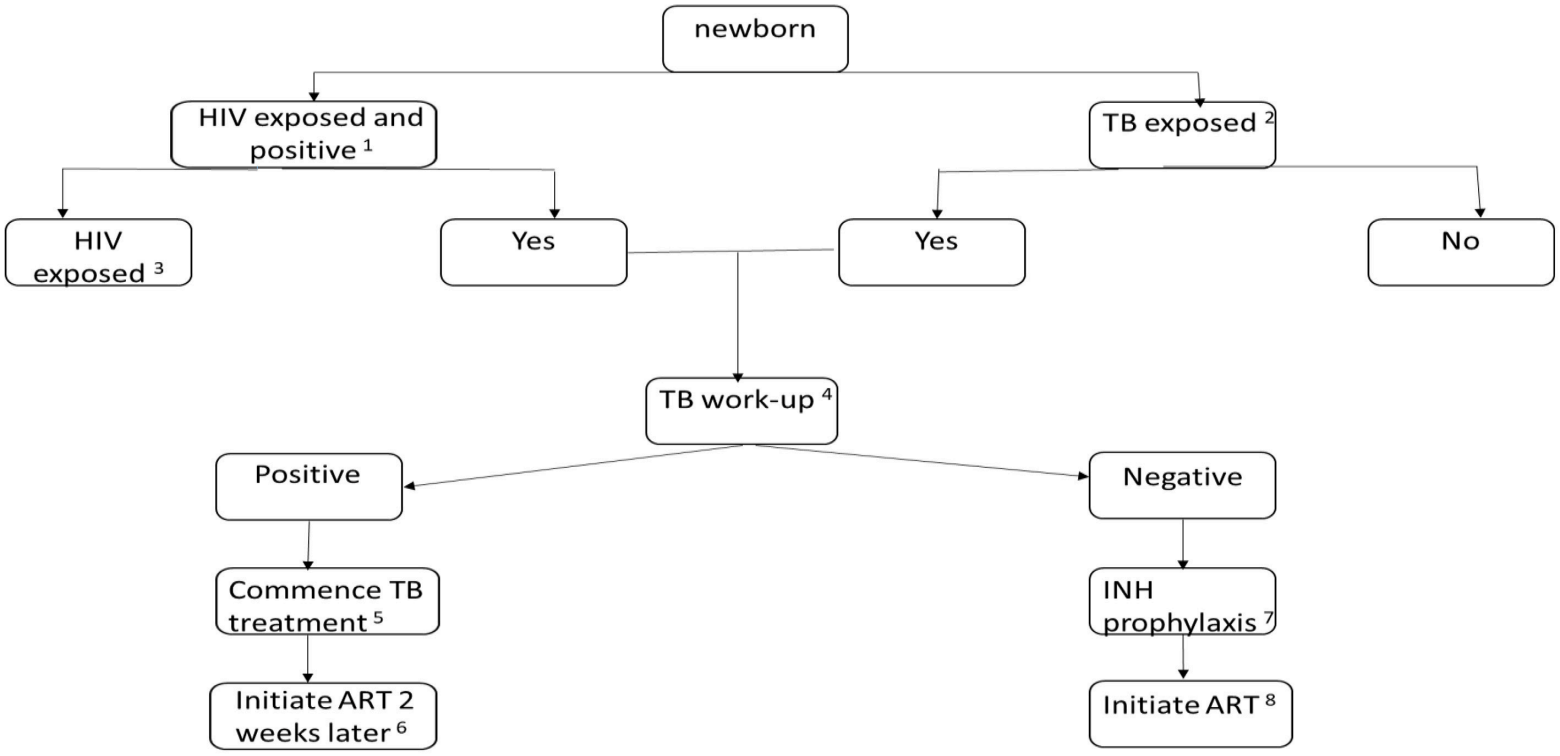
Algorithm for the evaluation and treatment of a newborn infant with TB +/- HIV exposure. (1) Early identification of HIV exposure (if not ascertained in the antenatal period) with maternal testing. Confirmation of transmission with PCR testing of the infant at 4–6 weeks. However, birth testing may be beneficial to early identification and treatment of perinatally infected infants. (2) Identify the risk of TB in the mother: thorough history, contact exposure, symptom screening, clinical examination, chest x-ray, sputum microscopy, culture and sensitivity and GeneXpert, if risk of miliary TB or TB meningitis is suspected: lumbar puncture, as necessary, initiate appropriate TB therapy in the mother. If the mother is diagnosed with TB, the placenta should be evaluated with histologic staining (including acid-fast bacilli) and tissue culture. Consider whether mother could have resistant TB. (3) If the newborn is HIV exposed, initiate prevention of mother to child prophylaxis: twice daily Zidovudine with added daily Nevirapine or Nevirapine + Lamivudine if there is a high risk of transmission. (4) The TB exposed newborn may present with symptoms or be asymptomatic. Clinical observation and examination for symptoms and signs forms part of the diagnostic process. Other investigations: chest radiograph, abdominal ultrasound, Tuberculin skin testing, specimens for TB culture and GeneXpert. (5) TB therapy for drug susceptible TB in the neonate: 4 months of daily: Rifampicin 15 mg/kg, Isoniazid 10 mg/kg, Pyrazinamide 35 mg/kg, Ethambutol 20 mg/kg, followed by 2 months of daily doses: Rifampicin 15 mg/kg, Isoniazid 10 mg/kg. (6) Initiate ART consisting of: Zidovudine, Lamivudine and Nevirapine or Raltegravir. (7) If confirmed or suspected drug sensitive TB disease is excluded, Isoniazid prophylaxis at 10 mg/kg should commence and continue for 6 months. Close monitoring and follow-up is required to identify TB symptoms and signs. (8) Initiate ART consisting of: Zidovudine, Lamivudine and Nevirapine or Raltegravir.

In Uganda, TB infection, measured by interferon gamma release assay (IGRA), was higher in HIV+ than uninfected mothers (59 vs. 42% *p* ≤ 0.001) with the odds of TB infection 21 times higher in HEU than HIV unexposed children <5 years (OR: 21.2, *P* = 0.008) (25). In South African neonates, 27 of 41 (66%) babies born to mothers on TB therapy were also HIV exposed (26), highlighting the concern of dual exposure in high prevalence settings. Neonates with maternal TB +/– HIV exposure should be examined and evaluated soon after birth (Figure 1).

## Impact of ART on TB Disease

TB treatment without ART results in suboptimal outcomes. In a South African study of ART naïve HIV/TB co infected children, 21% died during TB therapy with most deaths associated with advanced HIV disease (27). Of the 16 children who required a second course of TB therapy due to worsening or non-resolving TB disease, 43% (6/14) were persistently culture positive, emphasizing the importance of an immune response to cure TB (27). A study conducted pre- and post- the national ART rollout demonstrated a dramatic reduction in incident TB from 53/100-person years preceding the rollout to 6/100-person years thereafter, despite a persistently high rate of HIV/TB co-infection (28). Further, mortality was highest in those with TB prior to commencing ART (28). Similarly, a multicentre retrospective study reported a 70% reduction of incident TB after starting ART (29).

In the Children with HIV Early antiRetroviral (CHER) trial, TB was diagnosed less commonly in infants randomized to early ART commenced at a median age of 7.4 weeks than in the deferred arm where ART began at a median age of 6 months (8.3 vs. 20.2%: *P* = 0.014, Fisher's exact two-tail test, *post-hoc* analysis), in the first year of the trial. Infants were asymptomatic with CD4+ T-cell percentages ≥25% at study entry (30).

## BCG

Bacillus Calmette-Guérin (BCG) is currently the only available anti-TB vaccine. In use since 1921, the *M. bovis*-derived live attenuated vaccine is most effective in those with no prior exposure to mycobacteria (31). An observational study assessing BCG efficacy documented protection for up to 10 years when given in infancy and up to 20 years when first vaccinated at 10–15 years of age (32). Revaccination of school-aged children after infant vaccination shows little protection against TB disease (33). However, a recent study using interferon gamma release assays (IGRA) in adolescents, showed that BCG was effective in resolving TB infection (34). BCG is ineffective in HIV+, immune suppressed children who are at risk for disseminated BCG disease (35). Thus, the WHO recommends that in HIV+ infants, BCG should be administered only once ART has been initiated and the infant/child is immune competent (CD4% >25% in <5 years or CD4 count ≥200 in those ≥5 years) (35).

This recommendation implies early HIV testing and delaying BCG administration in HEU infants. After the adoption of early ART in infants from 6 weeks of age, disseminated BCG was no longer seen (Personal observation—MF Cotton). However, HIV+ infants not identified in infancy are at high risk for disseminated BCG. In South Africa, BCG induced immune responses at 52 weeks of age did not differ in HEU infants receiving the vaccine at birth compared to 14 weeks of age (36). A similar study is currently underway in Uganda (NCT02606526). With the worldwide acceptance of early infant HIV diagnosis at birth and immediate ART, BCG should continue to be given at birth as commonly practiced.

New TB vaccines for treatment, preventative pre-exposure prevention and post-treatment relapse prevention are under study (37). For HIV, given earlier ART initiation, these vaccines may have a role.

## TB Infection in the HIV+ or Exposed Child

“Latent TB infection” (LTBI) (i.e., *M.tb* infection without disease), usually indicated by a positive tuberculin skin test (TST) or IGRA is common in children. In the era preceding anti-TB therapy, 30–40% of infants with proof of infection and under a year of age developed pulmonary TB and 10–20% developed either miliary TB or TB meningitis (38). HIV infection and exposure without infection may increase this risk of progression to disease (Box 2). This could either be due to more TB in pregnancy or as a result of specific immune defects from HIV exposure *in utero* (39). Therefore, we consider the term “latent” a misnomer as it implies inactivity rather than preclinical disease with a strong likelihood of progression to disease.

Box 2 **Key facts.**

- HIV infection increases the risk of developing TB disease.
- Antiretroviral therapy dramatically reduces TB related mortality and morbidity in HIV/TB co-infection.
- Identification, screening, and early preventative therapy of pediatric household contacts is key in preventing progression of infection to disease.
- New child friendly TB and HIV drug formulations may improve tolerability, adherence, and treatment outcomes.
- Despite good outcomes, drug resistance remains a concern in childhood TB, particularly those with TB/HIV co-infection. Therefore, accessing drug sensitivity information from source cases and microbiological diagnosis are essential.

**Figure 2:**
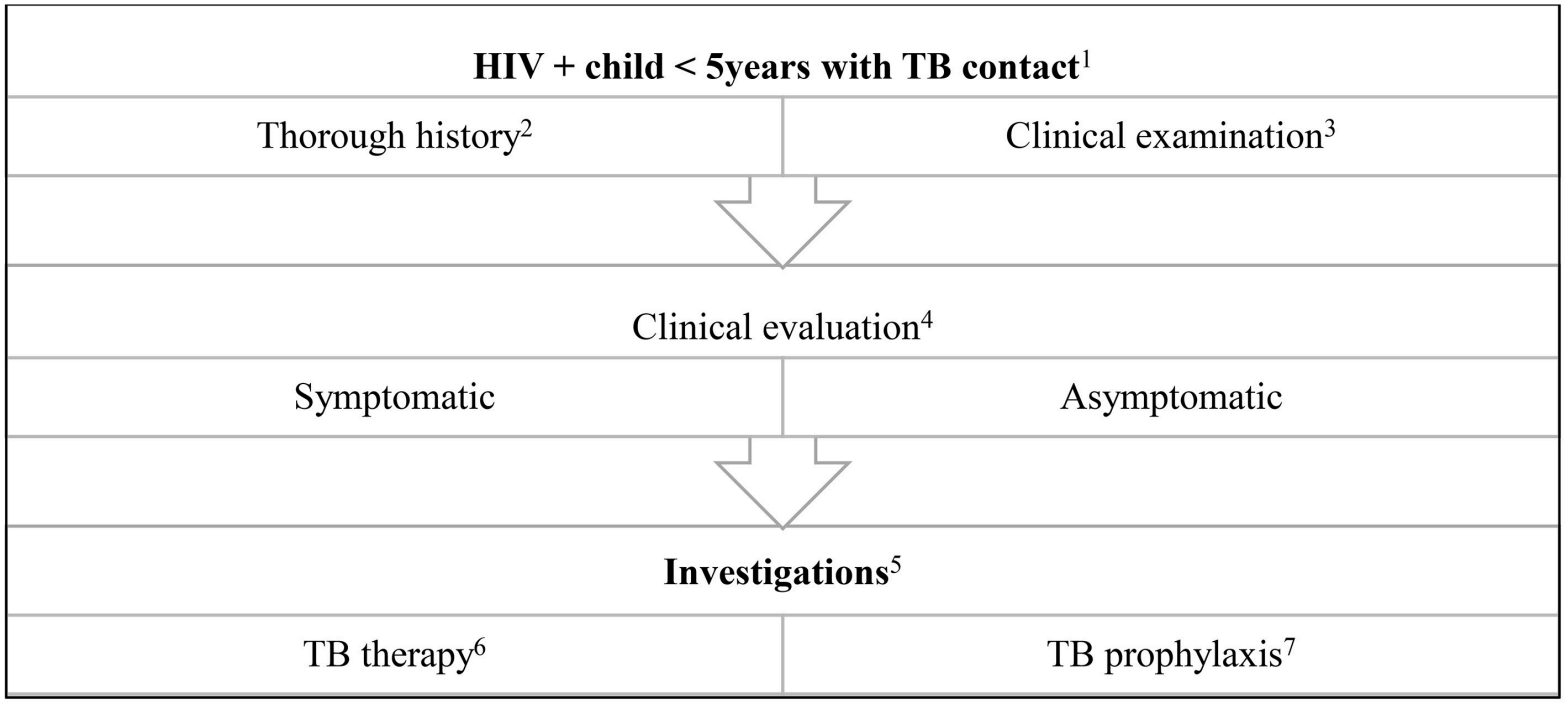
Algorithm for the evaluation and treatment of a child exposed to a TB source case. (1) Health promotion of testing all children in contact with an adult TB source case is important. Children ≤5 years of age are at high risk for TB disease. (2) History includes that of the source case: drug susceptible or drug resistant TB, compliance on TB treatment, first or second episode of TB. History of the child includes weight loss, loss of playfulness, coughing, fever, drenching night sweats. (3) Anthropometry is important in the clinical examination of all children. Respiratory signs may be non-specific and other HIV related respiratory conditions should be considered e.g., non-TB bacterial pneumonia, lymphocytic interstitial pneumonitis. (4) Children with a history of a TB contact may be asymptomatic, or present with non-specific symptoms for example, growth faltering. (5) These include a tuberculin skin test, chest radiograph and IGRA. In the symptomatic child, respiratory or other sampling for GeneXpert and microscopy (including acid-fast bacilli), culture and sensitivity, serum culture. Consider lumbar puncture and neuro imaging if suspicion of military TB or TB meningitis. Specialized tests indicated if extra pulmonary TB is suspected e.g., abdominal ultrasound, cardiac echo etc. (6) Uncomplicated TB: 4 months of daily Rifampicin 15 mg/kg, Isoniazid 10 mg/kg, Pyrazinamide 35 mg/kg followed by 2 months of daily Rifampicin 15 mg/kg, Isoniazid 10 mg/kg. In HIV+ children or complicated TB: 4 months of daily Rifampicin 15 mg/kg, Isoniazid 10 mg/kg, Pyrazinamide 35 mg/kg, Ethambutol 20 mg/kg, followed by 2 months of daily doses: Rifampicin 15 mg/kg, Isoniazid 10 mg/kg. (7) If TB has been clinically excluded, 6 months of Isoniazid prophylaxis is recommended. An alternative is 12 weekly doses of Isoniazid and Rifapentine.

After TB infection, there is an immune response preceding clinical disease. Identification and management during this phase prevents childhood disease and its consequences (Box 2). The WHO includes infants and children <5 years of age exposed to a bacteriologically confirmed pulmonary TB household source case and HIV+ people as high risk populations for treating and preventing TB (2). Additionally, in 2018, updated guidelines recommend testing and prevention treatment those ≥5 years of age in high incidence countries, where a bacteriologically confirmed PTB household source case was present (40). Immune based TB infection tests and preventative therapy are key components to manage TB infection prior to overt clinical disease (Figure 2).

TSTs are cost effective, easy to administer and are commonly used in risk stratification. The Mantoux TST is the preferred testing method in resource limited settings (41). However, a significant limitation is that sensitivity decreases with immune compromise and malnutrition, often prevalent co-morbidities in TB endemic areas, while specificity is reduced by cross reactivity with non-tuberculous mycobacteria (NTM) and BCG (42). In addition, apart from stock-outs, TST requires a cold chain. Despite having a low sensitivity in HIV+ children not on ART, it is still useful as a positive test confirms TB (43).

IGRA's induce a measurable *M.tb* specific T-cell immune response, overcoming cross reactivity with BCG and NTMs. Commonly used IGRA's include the QuantiFERON-TB gold In-Tube (QFT-IT, Qiagen Venlo, Netherlands)—a whole blood assay and the T-SPOT.TB (Oxford Immunotec, Oxford, United Kingdom), an enzyme-linked immunospot assay. These perform better than the TST in detecting *M.tb* infection (44). The WHO now recommends IGRA use in high burden settings if affordable, however the TST is adequate (41).

In a high burden TB and HIV setting, the QuantiFERON TB Gold in-Tube test performed better than the T-SPOT.TB test in detecting recent *M.tb* infection, regardless of HIV status in children. Both IGRAs performed better than TST (45).

Access to TB prevention therapy is dependent on stable national programs, trained staff and adequate resources. While testing for *M.tb* infection is highly recommended in HIV+ people and all children <5 years of age with a bacteriologically confirmed household contact, TB preventive therapy should be initiated even in the absence of TST or IGRA testing, once active TB has been excluded (40).

All HIV+ adults and children above 12 months of age require TB prevention therapy. Globally, the number of HIV+ adults accessing TB preventative treatment plateaued in 2014, while the number of children <5 years of age initiating this therapy continues to climb (3). In 2017, 67 countries initiated TB preventive treatment in more than 958,000 HIV+ patients, with two thirds also initiating ART. South Africa, the largest contributor, initiated ~370,000 HIV+ on TB preventative therapy, of whom 53% began ART. Additionally ~292,000 children <5 years of age accessed TB preventive therapy, a 3-fold rise from the ~87,000 in 2015 (3).

In otherwise healthy children, lengthy preventive therapy courses foster poor adherence (46). Daily isoniazid for 6 months is now only one of multiple options, allowing a tailored approach. New recommendations include either daily rifampicin and isoniazid or weekly isoniazid and rifapentine for 3 months (40). A large clinical trial in children aged 2–17 years reported that 12 weekly doses, of isoniazid + rifapentine was safe and non-inferior to isoniazid for 9 months; the completion rates being better with the shorter regimen (88 vs. 80% respectively, *p* = 0.003) (47). In HIV+ adults, the shorter regimen was safe, effective, better tolerated and with higher rates of treatment completion (48). To further facilitate adherence a pharmacokinetic study of a child friendly isoniazid/rifapentine fixed dose combination is planned for young HIV+ and HIV uninfected children below 12 years of age through the TB Trial Consortium.

## TB Diagnosis

Diagnosing childhood TB remains challenging. In TB/HIV endemic regions, the diagnostic conundrum is complicated by the overlapping, non-specific nature of clinical presentation of both diseases (Table 1).

In resource limited settings, a TB confirmed household contact, clinical symptoms and a suggestive chest radiograph all aid the decision to treat. In HIV+ children however, immune deficiency may alter the clinical presentation of TB disease and influences interpretation of x-rays due to non-TB, HIV-associated lung disorders such as lymphoid interstitial pneumonitis and bronchiectasis.

In a Cape Town study exploring the reliability of clinical signs and symptoms as a diagnostic tool, symptoms and risk were stratified. High risk was defined as <3 years of age or HIV+. Fatigue was the most sensitive presenting symptom in the low risk category (94.2%) and non-remitting cough in the high risk category (8.9% in <3 years, 100% in HIV +) (43). Combining presenting symptoms with objective weight loss is diagnostically accurate in low-risk children, [sensitivities of 82.3 and 51.8% (<3 years), 56.2 % (HIV+), respectively]. A high index of suspicion, positive contact history, obtaining the appropriate samples, and regular follow-up are the most important components for TB diagnosis in HIV+ children and very relevant regardless of HIV status.

Chest radiographs are useful for TB diagnosis. However, interpretation is challenging in the setting of TB/HIV co-infection. In a recent study of diagnostic accuracy of chest radiographs, sensitivity ranged from 61 to 94% and specificity from 20 to 48% in assessments by pediatric radiologists and pulmonologists (49). While the introduction of a chest radiograph reading and recording system showed minimal value (50), the use of digital chest radiographs with computer aided diagnosis may improve its diagnostic value, particularly in the asymptomatic contact and eliminating the need for specialist interpretation (51).

Point of care (PoC) tests improve case detection with rapid turn-around time. The WHO endorsed GeneXpert MTB/RIF® (Cepheid Inc., Sunnyvale, CA, United States) and recently the more sensitive GeneXpert MTB/RIF Ultra®. These automated cartridge-based nucleic acid amplification tests can detect *M.tb* and rifampicin resistance within 2 h (52). In South Africa, due to the need for regular device maintenance and good transport infrastructure, the GeneXpert test is usually performed in central laboratories. In a study of banked specimens from children, standard GeneXpert® detected 63.2%, Ultra® 73.7% with culture detecting 82.9% of childhood TB cases (53). Therefore, mycobacterial culture remains the “gold standard.” On the horizon is an Xpert panel to detect isoniazid, fluoroquinolone and aminoglycosides resistance in *M.tb* (54).

Microbiological diagnosis remains a key issue. Although induced sputum has better yields than gastric aspirates, it requires infrastructure and strict adherence to infection control to protect medical personnel. Nasopharyngeal aspirates, although easy to perform, also require respiratory precautions. Logistical issues with gastric aspirates include overnight fasting and skilled personnel to undertake the procedure (55). Obtaining multiple gastric aspirate specimens on a single day appears as good as daily specimens over 3 days (56).

Increasing attention is being given to stool collection. Although *M.tb* stool culture does not work well-due to contamination (57) GeneXpert shows promise but is less sensitive than for respiratory or gastric specimens (58, 59). The concordance of stool GeneXpert with bacteriologic confirmation was 31.9% but this test is extremely useful for severe TB, especially with cavitation on chest radiography (59).

Progress has been made on identifying immuno-metabolic signatures in children and adults for *M.tb* infection and TB disease irrespective of HIV status (60).

The advantages and disadvantages of TB diagnostic tools and the impact of HIV coinfection are summarized in Table 2 (61).

**Table 2:** Advantages and disadvantages of TB diagnostic modalities and the impact of HIV co-infection.

	**Advantages**	**Disadvantages**	**Impact of HIV co-infection**	**Improved diagnostic ability**
Clinical signs and symptoms	Easy to identify and elicit, useful for screening	Non-specific, may be asymptomatic	Overlapping signs and symptoms	Used with risk stratification, and the presence of a household contact
Chest radiograph	Available in resource limited settings, non-invasive investigation	Radiation exposure (though minimal), inter-reader variability	Distinguishing TB from HIV related pulmonary disease may be challenging	The use of computer aided diagnosis
Tuberculin skin testing	Easy to administer, point of care, confirms TB	Patient must return for reading and interpretation, does not distinguish between infection and disease	size of induration as a positive parameter differs Reduced sensitivity	
Immune based testing	Improved specificity and sensitivity over the TST	Laboratory based, does not distinguish between infection and disease		New generation interferon gamma release assay: QuantiFERON-TB Plus has novel CD8^+^ T-cell stimulating peptides to increase sensitivity when reduced immunity is present. More data is essential (61)
Microscopy	Direct observation confirms diagnosis	Paucity of disease impacts negatively on specificity		
Culture	Gold standard of diagnosis	Lengthy process, laboratory based		
GeneXpert MTB/RIF	Point of care, rapid diagnosis, identifies rifampicin resistance	Respiratory sampling is difficult	Decreased sensitivity in HIV coinfection	Use of alternative sampling i.e., stool The GeneXpert Ultra improves sensitivity in paucibacilliary disease and HIV coinfection Increased resistance profile

## Childhood TB Therapy and TB/HIV Co-Therapy

TB cure in children is multi-faceted. Treatment success is dependent on establishing therapeutic drug levels while avoiding adverse effects, maintaining good adherence, and preventing drug resistance. In the HIV/TB co-infected children, overcoming drug-drug interactions adds complexity to both TB cure and HIV suppression.

Development of child specific HIV and TB therapeutics often lags behind that in adults. Historically, extrapolating childhood anti-TB drug dosing from adult data has yielded sub-therapeutic serum drug levels (62). Children absorb and metabolize drugs differently to adults and require age-specific dosage adjustments.

For example, glucuronidation is reduced in neonates and expression of Cytochrome P450 enzymes changes with age (63). Additionally, liver clearance is more active in children than adults, thus children require higher dosages per kg (64). Higher mg/kg doses of first line drug susceptible TB treatment were recommended for children in 2010, approximately 40 years after their introduction (Table 3) (65). Despite demonstrating that these higher doses achieved adequate therapeutic serum drug levels (66), delivery involved either the adaption of the adult fixed dose combinations or combining the individual drugs. However, child-friendly fixed dose combinations (FDCs) containing rifampicin, isoniazid and pyrazinamide for children up to 25 kg have now been developed (67). To improve adherence, these FDCs are dispersible, palatable and dosage is by weight band (68). Soon, individual dispersible ethambutol and isoniazid tablets will also be introduced (68). A recent systematic review concluded that HIV infection may reduce exposure to first line anti-TB drugs and contribute to poorer treatment responses (69). However, because of much heterogeneity, firm conclusions could not be made. The authors recommended a consistent and homogeneous approach to studies and a uniform quality assessment tool for PK studies (69).

**Table 3:** Dosing, side effects and interaction for drug susceptible TB treatment.

**Name of medication**	**Dose**	**Available as fixed dose combination**	**Common side effects**	**Drug-drug interactions**
Rifampicin	15 mg/kg (10–20 mg/kg)	Yes	Gastrointestinal symptoms, hepatotoxicity, thrombocytopaenia, CNS disturbances	ARV's, esp. PI's, contraceptives, phenytoin, antifungals, fluoroquinolones
Isoniazid	10 mg/kg (7–15 mg/kg)	Yes	Neurotoxicity, peripheral neuritis, raised liver enzymes, anemia, thrombocytopaenia	Antiepileptics, benzodiazapines, theophylline, warfarin
Pyrazinamide	35 mg/kg (30–40 mg/kg)	Yes	Hepatotoxicity, arthralgia, myalgia, hypersensitivity reactions	Probenecid, allopurinol, colchicine, cyclosporine, may cause false urine ketone results
Ethambutol	20 mg/kg (15–25 mg/kg)	No	Optic neuritis, peripheral neuritis, raised liver enzymes, hypersensitivity	Aluminum hydroxide (antacids)

While shortening treatment duration may improve adherence, adult studies show an unacceptable risk of relapse (70). Thus, the standard 6 month therapy regimen (2 months intensive phase and 4 months continuation phase) is currently recommended for drug susceptible TB (71) (Figure 2). In India, a standard thrice weekly TB treatment regimen administered to HIV+ and uninfected children had a high rate of sub-therapeutic plasma rifampicin concentrations in both arms and poorer clinical outcomes (72). However, as many children have paucibacillary TB, mainly confined to intrathoracic lymph nodes and no cavities, shorter daily treatment may be possible and is currently being studied (73).

One of the challenges of treating TB/HIV co-infected children is overcoming drug-drug interactions of the rifamycins, with either non-nucleoside reverse transcriptase inhibitors (NNRTI's) or protease inhibitors (PI's) (Table 3). Rifampicin, an integral component in drug susceptible TB therapy, is a strong inducer of cytochrome P450 enzymes, in particular, CYP3A, increases breakdown of the NNRTI nevirapine (NVP) and the PI, ritonavir-boosted lopinavir (LPV/r). Rifampicin increases p-glycoprotein expression, promoting drug efflux from cells (74, 75).

Rifampicin can be used concomitantly with efavirenz, a commonly used NNRTI, in adults and children above 3 years of age and weight above 10 kg. Children require screening for G516T, the slow metabolism CYP 2B6 variant, common in Africa, which increases toxicity (76).

In the early years of the ART rollout, NVP was used commonly in much of Africa, co-formulated into a FDC with lamivudine and stavudine (77). Rifampicin significantly reduces the plasma concentrations and bioavailability of NVP in children (78–80). However, in adults increasing the dose of NVP from 200 to 300 mg produced adequate levels (81).

Ritonavir (RTV) co-formulated with LPV in a 1:4 ratio, inhibits CYP3A sufficiently to allow good LPV exposure. Lopinavir/ritonavir (LPV/r) is in first line ART in children <3 years of age (82). However, this protection is insufficient to overcome rifampicin-induced CYP3A induction. To overcome this effect, RTV is “super-boosted” to a 1:1 ratio with LPV. This strategy was first used in a small study of older children (74) and later confirmed in a larger study which included many infants under a year of age (83). However, barriers to super-boosting include a short shelf life of and need to refrigerate RTV solution. In addition, RTV has an unpleasant taste, which may jeopardize adherence.

A taste-masked solid formulation of LPV/r, lamivudine and abacavir is in development (84) and should improve tolerability remove barriers to storage. In addition, a solid formulation of ritonavir with a long shelf life will soon be available.

While “double dosing” of LPV/r gives adequate lopinavir exposure in adults (85), this strategy yields suboptimal levels in children (86). Pre-dose lopinavir concentrations were reduced by >80% in children on rifampicin (median 0.7 mg/l) compared with controls (4.2 mg/l; *P* < 0.001) and were below the minimum recommended concentration of 1 mg/l in 60 % (12/20) children with TB compared to 8% (2/24) of controls (*p* <0.001) (87).

While rifapentine, a long-acting rifamycin, suitable for weekly administration, is not yet approved for children, its potential for drug-drug interaction is almost that of rifampicin but can be used with EFV (88–90).

Integrase strand transfer inhibitors, both raltegravir (RGV) and dolutegravir (DTG) have been successfully used in adults requiring rifampicin, which potentially increases elimination through UDP-glucuronosyltransferase upregulation (91). For adults, although a single study suggests that dosage alteration is unnecessary (92), others recommend doubling the dose (93). For raltegravir, the twice daily dose is doubled and for dolutegravir, the daily dose is given twice daily (94). In children, preliminary RGV data supports doubling the twice daily dosage (95) and for DTG, studies of doubling the dose while on rifampicin are under way.

## Drug Resistant TB and HIV

Drug resistant TB is a major concern with an estimated 2 million children infected with multi-drug resistant (MDR) TB (Box 2) (96). In 2017, only 25% of the 558,000 MDR cases received appropriate therapy. In addition, in both incident cases and MDR TB in retreatment cases, primary transmission of the drug resistant strain is more likely than new resistance occurring on treatment (97). Exposure to a MDR TB source case has a higher risk of infection (aOR 2.05) but less risk of disease (aOR 0.43) than exposure drug susceptible *M.tb*, suggesting lower virulence for MDR *M.tb* (98). While similar results were found in Peru (99) and in India (100) confounders in these studies included population group ethnicities, differences in socio-economic status and length of exposure to the adult source case prior to enrolment (98). It is clear however, that both drug sensitive and resistant TB can cause severe disease and death.

The treatment of childhood MDR TB yields good outcomes in both HIV+ and uninfected children (101, 102). However, poor nutritional status and severity of disease contribute significantly to mortality and treatment failure (103). As with drug sensitive TB, ART is essential for MDR TB in HIV+ children (104). A standardized, shorter duration of treatment is successful for MDR TB in adults (105, 106), and the 9–12 month regimen is now recommended for those without prior exposure to second-line treatment agents or resistance to fluoroquinolones or second line injectables (107). While studies of shorter treatment regimens in children are lacking, the recommendation extends to all children.

As rifampicin is not used in MDR TB treatment, the main issues for ART are overlapping toxicity of anti-TB medications and emerging safety profiles of new anti-TB medications such as delaminid and bedaquiline which cause QT abnormalities. As LPV/r can cause conduction disturbances, heightened awareness is important. Most second-line anti-TB drugs, including the fluoroquinolones, have no clinically significant drug-drug interactions with ARVs and there is no evidence that HIV+ children have a higher risk of adverse events from MDR-TB infection treatment. However, ARVs and anti-TB medications have similar side effects.

Ethionamide, pyrazinamide, terizidone and NVP cause hepatotoxicity. As many TB regimens for drug resistance include 4–5 medications, adherence to both ART and anti-TB treatment may be difficult (108).

## The Immune Reconstitution Inflammatory Syndrome

The immune reconstitution inflammatory syndrome (IRIS) is well-described in HIV+ patients shortly after starting ART. It occurs most commonly in those with advanced disease. <underline >IRIS can occur with unnmasking of an infection or disease process not previously suspected or paradoxical if new inflammation of a known disease appears on ART (109). In children where neonatal<underline >bacille Calmette-Guerin (BCG) immunization is routine and TB is common, the most common forms of IRIS in children are due to BCG and TB (110). Both unmasking and paradoxical TB IRIS are described in children (111, 112). In the CHER trial, early ART was associated with a significant reduction in BCG IRIS adenitis provided that CD4 depletion had not yet occurred (113). Screening for TB pre-ART is important and includes a contact history for TB, chest X-ray, and mycobacterial culture if clinically suspected or if chest radiology is abnormal. Despite these investigations, unmasking TB IRIS can still occur. If there is no active TB disease and the child is over a year of age, isoniazid should be given for 6 months (40).

## Conclusion

TB/HIV co-infection poses a great threat to the WHO END TB strategy. The increased risk of TB infection in HIV+ individuals requires the early identification and treatment of both diseases effectively and to remove barriers to care. A high index of suspicion for TB should be maintained continuously in all HIV+ children.

While diagnosis and clinical care of children with HIV, TB and coinfection has improved over the last decade, much more work is needed to eradicate TB.

## Author Contributions

All authors listed have made a substantial, direct and intellectual contribution to the work, and approved it for publication.

### Conflict of Interest Statement

The authors declare that the research was conducted in the absence of any commercial or financial relationships that could be construed as a potential conflict of interest.

## References

[1] Daniel TM. The history of tuberculosis. Respir Med. (2006) 100:1862–70. 10.1016/j.rmed.2006.08.006 16949809

[2] World Health Organization Global Tuberculosis Report 2017 (2017).

[3] World Health Organization Global Tuberculosis Report 2018 (2018).

[4] Centers for Disease Control & Prevention (CDC) Pneumocystis pneumonia—Los Angeles. MMWR Morb Mortal Wkly Rep. (1981) 30:250–2. 6265753

[5] Centers for Disease Control (CDC) Unexplained immunodeficiency and opportunistic infections in infants–New York, New Jersey, California. MMWR Morb Mortal Wkly Rep. (1982) 31:665–7. 6819445

[6] Barré-Sinoussi F Chermann JC Rey F Nugeyre MT Chamaret S Gruest J. Isolation of a T-Lymphotropic Retrovirus from a patient at risk for acquired immune deficiency syndrome. Am Assoc Adv Sci. (1983) 220:868–71. 6189183 10.1126/science.6189183

[7] Mann JM Francis H Quinn T Asila PK Bosenge N Nzilambi N. Surveillance for aids in a central african city: Kinshasa, zaire. JAMA. (1986) 255:3255–9. 3012131

[8] Chretien J. Tuberculosis and HIV. The cursed duet. Bull Int Union Tuberc Lung Dis. (1990) 65:25–8. 2350606

[9] Venturini E Turkova A Chiappini E Galli L de Martino M Thorne C. Tuberculosis and HIV co-infection in children. BMC Infect Dis. (2014) 14:S5. 10.1186/1471-2334-14-S1-S5 24564453 PMC4016474

[10] AIDSinfo|UNAIDS Available online at: http://aidsinfo.unaids.org/ (accessed October 15, 2018).

[11] Martin DJ Sim JG Sole GJ Rymer L Shalekoff S van Niekerk AB. CD4+ lymphocyte count in African patients co-infected with HIV and tuberculosis. J Acquir Immune Defic Syndr Hum Retrovirol. (1995) 8:386–91. 10.1097/00042560-199504000-00010 7882104

[12] Geldmacher C Zumla A Hoelscher M. Interaction between HIV and *Mycobacterium tuberculosis*: HIV-1-induced CD4 T-cell depletion and the development of active tuberculosis. Curr Opin HIV AIDS. (2012) 7:268–75. 10.1097/COH.0b013e3283524e32 22495739

[13] Madhi SA Huebner RE Doedens L Aduc T Wesley D Cooper PA. HIV-1 co-infection in children hospitalised with tuberculosis in South Africa. Int J Tuberc Lung Dis. (2000) 4:448–54. 10815739

[14] Daniel OJ Adejumo OA Gidado M Abdur-Razzaq HA Jaiyesimi EO. HIV-TB co-infection in children: associated factors and access to HIV services in Lagos, Nigeria. Public Heal Action. (2015) 5:165–9. 10.5588/pha.15.0027 26399285 PMC4576771

[15] Jeena PM Pillay P Pillay T Coovadia HM. Impact of HIV-1 co-infection on presentation and hospital-related mortality in children with culture proven pulmonary tuberculosis in Durban, South Africa. Int J Tuberc Lung Dis. (2002) 6:672–8. 12150478

[16] Wiseman CA Gie RP Starke JR Schaaf HS Donald PR Cotton MF. A proposed comprehensive classification of tuberculosis disease severity in children. Pediatr Infect Dis J. (2012) 31:347–52. 10.1097/INF.0b013e318243e27b 22315002

[17] Stinson K Giddy J Cox V Burton R Ibeto M Cragg C Reflections on a decade of delivering PMTCT in Khayelitsha, South Africa. South Africa J HIV Med. (2013) 14:76–86. 10.4102/sajhivmed.v19i1.701

[18] Goga A Chirinda W Ngandu NK Ngoma K Bhardwaj S Feucht U Closing the gaps to eliminate mother-to-child transmission of HIV (MTCT) in South Africa: understanding MTCT case rates, factors that hinder the monitoring and attainment of targets, and potential game changers. Samj South African Med J. (2018) 108:S17–24. 10.7196/SAMJ.2018.v108i3.12817

[19] Goga A Jackson D Lombard C Ramokolo V Ngandu N Sherman G Highest risk of mother to child transmission of HIV or death in the first 6 months postpartum: results from 18 month follow-up of an HIV-exposed national cohort, South Africa. J Int AIDS Soc. (2016) 19:27–8.

[20] King CC Kourtis AP Persaud D Nelson JAE Ziemniak C Hudgens MG. Delayed HIV detection among infants exposed to postnatal antiretroviral prophylaxis during breastfeeding. AIDS. (2015) 29:1953–61. 10.1097/QAD.0000000000000794 26153671 PMC4665628

[21] Kourtis AP King CC Nelson J Jamieson DJ Van Der Horst C. Time of HIV diagnosis in infants after weaning from breast milk. AIDS. (2015) 29:1897–8. 10.1097/QAD.0000000000000796 26153670 PMC4665637

[22] Afran L Garcia Knight M Nduati E Urban BC Heyderman RS Rowland-Jones SL. HIV-exposed uninfected children: a growing population with a vulnerable immune system? Clin Exp Immunol. (2014) 176:11–22. 10.1111/cei.12251 24325737 PMC3958150

[23] Landes M van Lettow M Chan AK Mayuni I Schouten EJ Bedell RA. Mortality and health outcomes of HIV-exposed and unexposed children in a PMTCT cohort in Malawi. PLoS ONE. (2012) 7:e47337. 10.1371/journal.pone.0047337 23082157 PMC3474798

[24] Goetghebuer T Smolen KK Adler C Das J McBride T Smits G Initiation of anti-retroviral therapy before pregnancy reduces the risk of infection-related hospitalization in HIV-exposed uninfected infants born in a high-income country. Clin Infect Dis. (2019) 68:1193–203. 10.1093/cid/ciy673 30215689 PMC13375565

[25] Marquez C Chamie G Achan J Luetkemeyer AF Kyohere M Okiring J. Tuberculosis infection in early childhood and the association with HIV-exposure in HIV-uninfected children in rural Uganda. Pediatr Infect Dis J. (2016) 35:524–9. 10.1097/INF.0000000000001062 26771662 PMC4829461

[26] Bekker A Du Preez K Schaaf HS Cotton MF Hesseling AC. High tuberculosis exposure among neonates in a high tuberculosis and human immunodeficiency virus burden setting. Int J Tuberc Lung Dis. (2012) 16:1040–6. 10.5588/ijtld.11.0821 22691968

[27] Hesseling AC Westra AE Werschkull H Donald PR Beyers N Hussey GD. Outcome of HIV infected children with culture confirmed tuberculosis. Arch Dis Child. (2005) 90:1171–4. 10.1136/adc.2004.070466 15964862 PMC1720190

[28] Walters E Cotton MF Rabie H Schaaf HS Walters LO Marais BJ. Clinical presentation and outcome of Tuberculosis in Human Immunodeficiency Virus infected children on anti-retroviral therapy. BMC Pediatr. (2008) 8:1. 10.1186/1471-2431-8-1 18186944 PMC2246130

[29] Martinson NA Moultrie H van Niekerk R Barry G Coovadia A Cotton M. HAART and risk of tuberculosis in HIV-infected South African children: a multi-site retrospective cohort. Int J Tuberc Lung Dis. (2009) 13:862–7. 19555536 PMC6374123

[30] Violari A Cotton M. Early antiretroviral therapy and mortality among HIV-infected infants. N Engl J Med. (2008) 359:2233–44. 10.1056/NEJMoa0800971 19020325 PMC2950021

[31] Mangtani P Abubakar I Ariti C Beynon R Pimpin L Fine PEM. Protection by BCG vaccine against tuberculosis: a systematic review of randomized controlled trials. Clin Infect Dis. (2014) 58:470–80. 10.1093/cid/cit790 24336911

[32] Mangtani P Nguipdop-Djomo P Keogh RH Trinder L Smith PG Fine PEM. Observational study to estimate the changes in the effectiveness of bacillus calmette-guérin (BCG) vaccination with time since vaccination for preventing tuberculosis in the UK. Health Technol Assess. (2017) 21:5–53. 10.3310/hta21390 28738015 PMC5534974

[33] Barreto ML Pereira SM Pilger D Cruz AA Cunha SS Sant'anna C. Evidence of an effect of BCG revaccination on incidence of tuberculosis in school-aged children in Brazil: second report of the BCG-REVAC cluster-randomised trial. Vaccine. (2011) 29:4875–7. 10.1016/j.vaccine.2011.05.023 21616115

[34] Nemes E Geldenhuys H Rozot V Rutkowski KT Ratangee F Bilek N Prevention of *M. tuberculosis* Infection with H4:IC31 Vaccine or BCG Revaccination. N Engl J Med. (2018) 379:138–49. 10.1056/NEJMoa1714021 29996082 PMC5937161

[35] World Health Organization BCG Vaccines: WHO Position Paper. Weekly Epidemiological Record Relevé Épidémiologique Hebdomadaire. (2018). p. 201–20.

[36] Hesseling AC Jaspan HB Black GF Nene N Walzl G Immunogenicity of BCG in HIV-exposed and non-exposed infants following routine birth or delayed vaccination. Int J Tuberc Lung Dis. (2015) 19:454–62. 10.5588/ijtld.14.0608 25860002 PMC4530999

[37] Kaufmann SHE Lange C Rao M Balaji KN Lotze M Schito M. Progress in tuberculosis vaccine development and host-directed therapies-a state of the art review. Lancet Respir Med. (2014) 2:301–20. 10.1016/S2213-2600(14)70033-5 24717627

[38] Marais BJ Gie RP Schaaf HS Hesseling AC Obihara CC Nelson LJ. The natural history of childhood intra-thoracic tuberculosis: a critical review of literature from the pre-chemotherapy era B. Int J Tuberc Lung Dis. (2004) 8:392–402. 15141729

[39] Ruck C Reikie BA Marchant A Kollmann TR Kakkar F. Linking susceptibility to infectious diseases to immune system abnormalities among HIV-exposed uninfected infants. Front Immunol. (2016) 7:1–12. 10.3389/fimmu.2016.00310 27594857 PMC4990535

[40] World Health Organization Latent Tuberculosis Infection: Updated and Consolidated Guidelines for Programmatic Management (2018). 30277688

[41] World Health Organization. Guidelines on the Management of Latent Tuberculosis Infection (2015). 25973515

[42] Petrucci R Lombardi G Corsini I Bacchi Reggiani ML Visciotti F Bernardi F. Quantiferon-TB gold in-tube improves tuberculosis diagnosis in children. Pediatr Infect Dis J. (2017) 36:44–9. 10.1097/INF.0000000000001350 27749653

[43] Marais BJ Gie RP Hesseling AC Schaaf HS Lombard C Enarson DA. A refined symptom-based approach to diagnose pulmonary tuberculosis in children. Pediatrics. (2006) 118:e1350–9. 10.1542/peds.2006-0519 17079536

[44] Connell TG Ritz N Paxton GA Buttery JP Curtis N Ranganathan SC. A three-way comparison of tuberculin skin testing, QuantiFERON-TB gold and T-SPOT.TB in children. PLoS ONE. (2008) 3:e2624. 10.1371/journal.pone.0002624 18612425 PMC2440545

[45] Mandalakas AM Kirchner HL Walzl G Gie RP Schaaf HS Cotton MF. Optimizing the detection of recent tuberculosis infection in children in a high tuberculosis-HIV burden setting. Am J Respir Crit Care Med. (2015) 191:820–30. 10.1164/rccm.201406-1165OC 25622087 PMC4407483

[46] Marais BJ. Improving access to tuberculosis preventive therapy and treatment for children. Int J Infect Dis. (2017) 56:122–5. 10.1016/j.ijid.2016.12.015 27993688

[47] Villarino ME Scott NA Weis SE Weiner M Conde MB Jones B. Treatment for preventing tuberculosis in children and adolescents: a randomized clinical trial of a 3-month, 12-dose regimen of a combination of rifapentine and Isoniazid. JAMA Pediatr. (2015) 169:247–55. 10.1001/jamapediatrics.2014.3158 25580725 PMC6624831

[48] Sterling TR Scott NA Miro JM Calvet G La Rosa A Infante R. Three months of weekly rifapentine and isoniazid for treatment of *Mycobacterium tuberculosis* infection in HIV-coinfected persons. AIDS. (2016) 30:1607–15. 10.1097/QAD.0000000000001098 27243774 PMC4899978

[49] Berteloot L Marcy O Nguyen B Ung V Tejiokem M Nacro B. Value of chest X-ray in TB diagnosis in HIV-infected children living in resource-limited countries: the ANRS 12229-PAANTHER 01 study. Int J Tuberc Lung Dis. (2018) 22:844–50. 10.5588/ijtld.18.0122 29991391

[50] Kosack CS Spijker S Halton J Bonnet M Nicholas S Chetcuti K. Evaluation of a chest radiograph reading and recording system for tuberculosis in a HIV-positive cohort. Clin Radiol. (2017) 72:519.e1. 10.1016/j.crad.2017.01.008 28236438

[51] Muyoyeta M Kasese NC Milimo D Mushanga I Ndhlovu M Kapata N. Digital CXR with computer aided diagnosis versus symptom screen to define presumptive tuberculosis among household contacts and impact on tuberculosis diagnosis. BMC Infect Dis. (2017) 17:301. 10.1186/s12879-017-2388-7 28438139 PMC5402643

[52] Chakravorty S Marie Simmons A Rowneki M Parmar H Cao Y Ryan J. The new Xpert MTB/RIF Ultra: improving detection of *Mycobacterium tuberculosis* and resistance to rifampin in an assay suitable for point-of-care testing. mBio. (2017) 8:e00812-17. 10.1128/mBio.00812-17 28851844 PMC5574709

[53] Nicol MP Workman L Prins M Bateman L Ghebrekristos Y Mbhele S. Accuracy of Xpert MTB/RIF Ultra for the diagnosis of pulmonary tuberculosis in children. Pediatr Infect Dis J. (2018) 37:e261–3. 10.1097/INF.0000000000001960 29474257

[54] Chakravorty S Roh SS Glass J Smith LE Simmons AM Lund K. Detection of isoniazid-, fluoroquinolone-, amikacin-, and kanamycin-resistant tuberculosis in an automated, multiplexed 10-color assay suitable for point-of-care use. J Clin Microbiol. (2017) 55:183–98. 10.1128/JCM.01771-16 27807153 PMC5228229

[55] WHO Guidance for National Tuberculosis Programmes on the Management of Tuberculosis in Children (2006).

[56] Al-Aghbari N Al-Sonboli N Yassin MA Coulter JBS Atef Z Al-Eryani A. Multiple sampling in one day to optimize smear microscopy in children with tuberculosis in Yemen. PLoS ONE. (2009) 4: e5140. 10.1371/journal.pone.0005140 19357770 PMC2663055

[57] Walters E Demers A-M van der Zalm MM Whitelaw A Palmer M Bosch C. Stool culture for the diagnosis of pulmonary tuberculosis in children. J Clin Microbiol. (2017) 55:3355–65. 10.1128/JCM.00801-17 28904186 PMC5703802

[58] Nicol MP Spiers K Workman L Isaacs W Munro J Black F. Xpert MTB/RIF testing of stool samples for the diagnosis of pulmonary tuberculosis in children. Clin Infect Dis. (2013) 57:e18–21. 10.1093/cid/cit230 23580738 PMC3703104

[59] Walters E van der Zalm MM Palmer M Bosch C Demers A-M Draper H. Xpert MTB/RIF on stool is useful for the rapid diagnosis of tuberculosis in young children with severe pulmonary disease. Pediatr Infect Dis J. (2017) 36:837–43. 10.1097/INF.0000000000001563 28151842 PMC5558052

[60] Bah SY Forster T Dickinson P Kampmann B Ghazal P. Meta-analysis identification of highly robust and differential immune-metabolic signatures of systemic host response to acute and latent tuberculosis in children and adults. Front Genet. (2018) 9:457. 10.3389/fgene.2018.00457 30337941 PMC6180280

[61] Horne DJ Jones BE Kamada A Fukushima K Winthrop KL Siegel SAR. Multicenter study of QuantiFERON®-TB gold plus in patients with active tuberculosis. Int J Tuberc Lung Dis. (2018) 22:617–21. 10.5588/ijtld.17.0721 29862944

[62] Burman WJ Cotton MF Gibb DM Walker AS Vernon AA Donald PR. Ensuring the involvement of children in the evaluation of new tuberculosis treatment regimens. PLoS Med. (2008) 5:e176. 10.1371/journal.pmed.0050176 18715115 PMC2517617

[63] Kearns GL Abdel-Rahman SM Alander SW Blowey DL Leeder JS Kauffman RE Developmental pharmacology—drug disposition, action, and therapy in infants and children. N Engl J Med. (2003) 12:349 10.1056/NEJMra035092 13679531

[64] Crom WR Relling MV Christensen ML Rivera GK Evans WE. Age-related differences in hepatic drug clearance in children: studies with lorazepam and antipyrine. Clin Pharmacol Ther. (1991) 50:132–40. 10.1038/clpt.1991.117 1868674

[65] World Health Organization Rapid Advice: Treatment of Tuberculosis Infection in Children (2010) 26269860

[66] Thee S Seddon JA Donald PR Seifart HI Werely CJ Hesseling AC. Pharmacokinetics of isoniazid, rifampin,and pyrazinamide in children younger than two years of age with tuberculosis: evidence for implementation of revised World Health Organization recommendations. Antimicrob Agents Chemother. (2011) 55:5560–7. 10.1128/AAC.05429-11 21968358 PMC3232790

[67] World Health Organization Technical Step Process to Switch to New Paediatric Tuberculosis Formulations (2016).

[68] UNICEF, World Health Organization FDC for TB Treatment in Children (2017).

[69] Daskapan A Idrus LR Postma MJ Wilffert B Kosterink JGW Stienstra Y. A systematic review on the effect of HIV infection on the pharmacokinetics of first-line tuberculosis drugs. Clin Pharmacokinet. (2018). [Epub ahead of print]. 10.1007/s40262-018-0716-8 30406475 PMC7019645

[70] Wallis RS Maeurer M Mwaba P Chakaya J Rustomjee R Migliori GB. Tuberculosis—advances in development of new drugs, treatment regimens, host-directed therapies, and biomarkers. Lancet Infect Dis. (2018) 16:e34–46. 10.1016/S1473-3099(16)00070-0 27036358

[71] World Health Organization Treatment of Tuberculosis: Guidelines for Treatment of Drug-Susceptible Tuberculosis and Patient Care (2017).

[72] Ramachandran G Kumar AKH Kannan T Bhavani PK Kumar SR Gangadevi NP. Low Serum concentrations of rifampicin and pyrazinamide associated with poor treatment outcomes in children with tuberculosis related to HIV status. Pediatr Infect Dis J. (2016) 35:530–4. 10.1097/INF.0000000000001069 26825153

[73] Chabala C Turkova A Thomason MJ Wobudeya E Hissar S Mave V. Shorter treatment for minimal tuberculosis (TB) in children (SHINE): a study protocol for a randomised controlled trial. Trials. (2018) 19:237. 10.1186/s13063-018-2608-5 29673395 PMC5909210

[74] Ren Y Nuttall JJC Egbers C Eley BS Meyers TM Smith PJ. Effect of rifampicin on lopinavir pharmacokinetics in HIV-infected children with tuberculosis. J Acquir Immune Defic Syndr. (2008) 47:566–9. 10.1097/QAI.0b013e31819c33a3 18197120

[75] Williamson B Dooley KE Zhang Y Back DJ Owen A. Induction of influx and efflux transporters and cytochrome P450 3A4 in primary human hepatocytes by rifampin, rifabutin, and rifapentine. Antimicrob Agents Chemother. (2013) 57:6366–9. 10.1128/AAC.01124-13 24060875 PMC3837889

[76] Moore CB Capparelli EV Samson P Bwakura-Dangarembizi M Jean-Philippe P Worrell C CYP2B6 genotype-directed dosing is required for optimal efavirenz exposure in children 3-36 months with HIV infection. AIDS. (2017) 31:1129–36. 10.1097/QAD.0000000000001463 28323755 PMC5623109

[77] World Health Organization Antiretroviral Therapy of HIV Infection in Infants and Children in Resource-Limited Settings: Towards Universal Access. Recommendations for a Public Health Approach. (2006). p. 171. 23741772

[78] Ribera E Pou L Lopez RM Crespo M Falco V Ocaña I. Pharmacokinetic interaction between nevirapine and rifampicin in HIV-infected patients with tuberculosis. J Acquir Immune Defic Syndr. (2001) 28:450–3. 10.1097/00042560-200112150-00007 11744833

[79] Cohen K Van Cutsem G Boulle A Mcilleron H Goemaere E Smith PJ. Effect of rifampicin-based antitubercular therapy on nevirapine plasma concentrations in South African adults with HIV-associated tuberculosis. J Antimicrob Chemother. (2008) 61:389–93. 10.1093/jac/dkm484 18096560

[80] Oudijk JM McIlleron H Mulenga V Chintu C Merry C Walker AS Cook A Gibb DM Burger DM. Pharmacokinetics of nevirapine in HIV-infected children under 3 years on rifampicin-based antituberculosis treatment. AIDS. (2012) 26:1523–8. 10.1097/QAD.0b013e3283550e20 22546991

[81] Ramachandran G Hemanthkumar AK Rajasekaran S Padmapriyadarsini C Narendran G Sukumar B. Increasing nevirapine dose can overcome reduced bioavailability due to rifampicin coadministration. J Acquir Immune Defic Syndr. (2006) 42:36–41. 10.1097/01.qai.0000214808.75594.73 16639340

[82] Violari A Lindsey JC Hughes MD Mujuru HA Barlow-Mosha L Kamthunzi P. Nevirapine versus ritonavir-boosted lopinavir for HIV-infected children. N Engl J Med. (2012) 366:2380–9. 10.1056/NEJMoa1113249 22716976 PMC3443859

[83] Rabie H Denti P Lee J Masango M Coovadia A Pillay S. Lopinavir–ritonavir super-boosting in young HIV-infected children on rifampicin-based tuberculosis therapy compared with lopinavir–ritonavir without rifampicin: a pharmacokinetic modelling and clinical study. Lancet HIV. (2018) 6:e32–42. 10.1016/S2352-3018(18)30293-5 30529029

[84] DNDi ‘4-in-1' LPV/r/ABC/3TC – DNDi. Available online at: https://www.dndi.org/diseases-projects/portfolio/two-4-in-1-lpv-r-based-fixed-dose-combinations/ (accessed November 14, 2018).

[85] La Porte CJL Colbers EPH Bertz R Voncken DS Wikstrom K Boeree MJ. Pharmacokinetics of adjusted-dose lopinavir-ritonavir combined with rifampin in healthy volunteers. Antimicrob Agents Chemother. (2004) 48:1553–60. 10.1128/AAC.48.5.1553-1560.2004 15105105 PMC400571

[86] Zhang C McIlleron H Ren Y Van Der Walt JS Karlsson MO Simonsson USH. Population pharmacokinetics of lopinavir and ritonavir in combination with rifampicin-based antitubercular treatment in HIV-infected children. Antivir Ther. (2012) 17:25–33. 10.3851/IMP1915 22267466 PMC3743021

[87] McIlleron H Ren Y Nuttall J Fairlie L Rabie H Cotton M. Lopinavir exposure is insufficient in children given double doses of lopinavir/ritonavir during rifampicin-based treatment for tuberculosis. Antivir Ther. (2011) 16:417–21. 10.3851/IMP1757 21555825

[88] Podany AT Bao Y Swindells S Chaisson RE Andersen JW Mwelase T. Efavirenz pharmacokinetics and pharmacodynamics in HIV-infected persons receiving rifapentine and isoniazid for tuberculosis prevention. Clin Infect Dis. (2015) 61:1322–7. 10.1093/cid/civ464 26082504 PMC4583578

[89] Weiner M Egelund EF Engle M Kiser M Prihoda TJ Gelfond JAL. Pharmacokinetic interaction of rifapentine and raltegravir in healthy volunteers. J Antimicrob Chemother. (2014) 69:1079–85. 10.1093/jac/dkt483 24343893 PMC4014856

[90] Farenc C Doroumian S Cantalloube C Perrin L Esposito V Cierien-Puiseux I Rifapentine once-weekly dosing effect on efavirenz, emtricitabine and tenofovir PKs. In: Conference, 233–4. Available online at: http://www.croiconference.org/sites/all/abstracts/493.pdf (accessed November 14, 2018).

[91] Maartens G Boffito M Flexner CW. Compatibility of next-generation first-line antiretrovirals with rifampicin-based antituberculosis therapy in resource limited settings. Curr Opin HIV AIDS. (2017) 12:355–8. 10.1097/COH.0000000000000376 28403028

[92] Grinsztejn B De Castro N Arnold V Veloso VG Morgado M Pilotto JH. Raltegravir for the treatment of patients co-infected with HIV and tuberculosis (ANRS 12 180 Reflate TB): a multicentre, phase 2, non-comparative, open-label, randomised trial. Lancet Infect Dis. (2014) 14:459–67. 10.1016/S1473-3099(14)70711-X 24726095

[93] Klis S Daskapan A Akkerman OW Alffenaar JW Stienstra Y. Raltegravir and rifampicin in patients with HIV and tuberculosis. Lancet Infect Dis. (2014) 14:1046–7. 10.1016/S1473-3099(14)70977-6 25444405

[94] Dooley K Kaplan R Mwelase T Grinsztejn B Ticona E Lacerda M Safety and efficacy of dolutegravir-based ART in TB/HIV co-infected adults at week 48. Oral abstract TUAB0206 in AIDS 2018. Available online at: https://programme.aids2018.org/Abstract/Abstract/6122 (accessed November 14, 2018).

[95] Meyers T Krogstad P Samson P Acosta E Moye J Townley E P1101: PhaseI/II study of raltegravir containing in HIV-TB cotreated children. Top Antivir Med. (2018) 26(Suppl 1):378s–9.

[96] Dodd PJ Sismanidis C Seddon JA. Global burden of drug-resistant tuberculosis in children: a mathematical modelling study. Lancet Infect Dis. (2016) 16:1193–201. 10.1016/S1473-3099(16)30132-3 27342768

[97] Kendall EA Fofana MO Dowdy DW. Burden of transmitted multidrug resistance in epidemics of tuberculosis: a transmission modelling analysis. Lancet Respir Med. (2015) 3:963–72. 10.1016/S2213-2600(15)00458-0 26597127 PMC4684734

[98] Golla V Snow K Mandalakas AM Schaaf HS Du Preez K Hesseling AC The impact of drug resistance on the risk of tuberculosis infection and disease in child household contacts: a cross sectional study. BMC Infect Dis. (2017) 17:593 10.1186/s12879-017-2806-x 28851285 PMC5576070

[99] Soto E Castro B Lopez S Coronel J Castillo E Alarcon V. Transmission of multidrug-resistant and drug-susceptible tuberculosis within households: a prospective cohort study. PLoS Med. (2015) 12:e1001843. 10.1371/journal.pmed.1001843 26103620 PMC4477882

[100] Radhakrishnan S Subramani R Risk of tuberculosis among contacts of isoniazid-resistant and isoniazid-susceptible cases. Int J Tuberc Lung Dis. (2011) 15:782–8. 10.5588/ijtld.09.0327 21575299

[101] Isaakidis P Casas EC Das M Tseretopoulou X Ntzani EE Ford N. Treatment outcomes for HIV and MDR-TB co-infected adults and children: systematic review and meta-analysis. Int J Tuberc Lung Dis. (2015) 19:969–78. 10.5588/ijtld.15.0123 26162364

[102] Ettehad D Schaaf HS Seddon JA Cooke GS Ford N. Treatment outcomes for children with multidrug-resistant tuberculosis: a systematic review and meta-analysis. Lancet Infect Dis. (2012) 12:449–56. 10.1016/S1473-3099(12)70033-6 22373593

[103] Chiang SS Starke JR Miller AC Cruz AT Del Castillo H Valdivia WJ. Baseline predictors of treatment outcomes in children with multidrug-resistant tuberculosis: a retrospective cohort study. Clin Infect Dis. (2016) 63:1063–71. 10.1093/cid/ciw489 27458026

[104] Harausz EP Garcia-prats AJ Law S Schaaf HS Kredo T Seddon JA. Treatment and outcomes in children with multidrug-resistant tuberculosis: a systematic review and individual patient data meta-analysis. PLoS Med. (2018) 15:1–27. 10.1371/journal.pmed.1002591 29995958 PMC6040687

[105] Van Deun A Maug AKJ Salim MAH Das PK Sarker MR Daru P. Short, highly effective, and inexpensive standardized treatment of multidrug-resistant tuberculosis. Am J Respir Crit Care Med. (2010) 182:684–92. 10.1164/rccm.201001-0077OC 20442432

[106] M Aung KJ Van Deun A Declercq E Sarker MR Das PK Hossain MA Successful “9-month Bangladesh regimen” for multidrug-resistant tuberculosis among over 500 consecutive patients. Int J Tuberc Lung Dis. (2014) 18:1180–7. 10.5588/ijtld.14.0100 25216831

[107] World Health Organization Global Tuberculosis Programme. WHO Treatment Guidelines for Drug-Resistant Tuberculosis: 2016 Update. (2016). p. 56. 27748093

[108] Cruz AT Garcia-Prats AJ Furin J Seddon JA Treatment of multidrug-resistant tuberculosis infection in children. Pediatr Infect Dis J. (2018) 37:1 10.1097/INF.0000000000002087 29742640

[109] Shelburne SA Hamill RJ Rodriguez-Barradas MC Greenberg SB Atmar RL Musher DW. Immune reconstitution inflammatory syndrome: emergence of a unique syndrome during highly active antiretroviral therapy. Medicine. (2002) 81:213–27. 10.1097/00005792-200205000-00005 11997718

[110] Smith K Kuhn L Coovadia A Meyers T Hu CC Reitz C. Immune reconstitution inflammatory syndrome among HIV-infected South African infants initiating antiretroviral therapy. AIDS. (2009) 23:1097–107. 10.1097/QAD.0b013e32832afefc 19417581 PMC2810152

[111] Rabie H Lomp A Goussard P Nel E Cotton M. Paradoxical tuberculosis associated immune reconstitution inflammatory syndrome presenting with chylous ascites and chylothorax in a HIV-1 infected child. J Trop Pediatr. (2010) 56:355–8. 10.1093/tropej/fmp141 20100782

[112] Zampoli M Kilborn T Eley B. Tuberculosis during early antiretroviral-induced immune reconstitution in HIV-infected children. Int J Tuberc Lung Dis. (2007) 11:417–23. 17394688

[113] Rabie H Violari A Duong T Madhi SA Josipovic D Innes S. Early antiretroviral treatment reduces risk of bacille Calmette- Guérin immune reconstitution adenitis. Int J Tuberc Lung Dis. (2011) 15:1194–200. 10.5588/ijtld.10.0721 21943845 PMC3183835

